# Extrapolation of in vitro effect concentrations to in vivo bioavailable concentrations using PBK modelling in humans for two classes of persistent and mobile compounds: triazoles and triazines

**DOI:** 10.1007/s00204-025-04268-w

**Published:** 2026-02-17

**Authors:** Abishek Laxmanan Ravi Shankar, Jenny Irwan, Max Spaenig, Maxim Carlier, Tanja Hansen, Nicole Zumbülte, Barira Islam, Patrik Lundquist, Richard Svensson, Todd Gouin, Timo Hamers, Sylvia E. Escher

**Affiliations:** 1 Fraunhofer Institute for Toxicology and Experimental Medicine, Nikolai- Fuchs-22 Strasse-1, 30625 Hannover, Germany; 2 Amsterdam Institute for Life and Environment, Section Environment and Health, Vrije Universiteit Amsterdam, 1081 HV Amsterdam, The Netherlands; 3 TZW: DVGW-Technologiezentrum Wasser (German Water Centre), Karlsruher Straße 84, 76139 Karlsruhe, Germany; 4 Certara Predictive Technologies, Sheffield, United Kingdom; 5 Department of Pharmacy, Uppsala University, Uppsala, Sweden; 6 TG Environmental Research, Sharnbrook, United Kingdom

**Keywords:** NAMs – new approach methodologies, PBK – physiologically based kinetic model, Triazines, Triazoles, QIVIVE – quantitative in vitro to in vivo extrapolation, RAX - read-across approach, ADME – absorption, distribution, metabolism, excretion

## Abstract

Bottom-up in vitro ADME parameterized PBK models play a vital role in Next Generation Risk Assessment (NGRA), which evaluates the toxicological hazards of compounds through New Approach Methods (NAMs). The ZeroPM project, funded by the EU under Horizon 2020, develops strategies to prioritize persistent (P) and mobile (M) compounds for regulatory measures based on exposure levels and toxicological properties. This study developed a quantitative in vitro to in vivo extrapolation (QIVIVE) approach for two classes of PM compounds – 9 triazines and 16 triazoles. The virtual in vitro distribution model (VIVD) derived free medium benchmark concentration values (EC20 and IR1.5 (mol/L)) from a previously published NAMs testing battery. The VIVD model accounted for partitioning and ionization of PM compounds, while a bottom-up PBK model using in vitro ADME data accurately predicted rat plasma concentrations for tebuconazole and cyromazine. The same assumptions were translated to a human oral-route PBK model. Most triazines/triazoles had in vitro ADME data, where the data gaps were filled using read-across approach. The human PBK model employed forward dosimetry to estimate plasma concentrations from preclinical lowest observed adverse effect levels (LOAELs) or hypothetical exposure data, using surface- and groundwater concentrations as worst-case scenarios. Comparison to QIVIVE data indicates that NAMs-derived values are protective and can serve as basis for human risk assessment or risk-based prioritization.

**Supplementary Information:**

The online version contains supplementary material available at 10.1007/s00204-025-04268-w.

## Introduction

Persistent (P) and mobile (M) compounds are concerning for the human health and for the environment due to their long half-lives in the environment and potential accumulation in drinking water (Neumann and Schliebner 2017). A substance is classified as persistent if its degradation half-life exceeds 40–180 days in marine, fresh, or estuarine water. It is classified as “very persistent” (vP) if the half-life exceeds 180 days (Neumann and Schliebner 2017). Mobility is assessed for compounds meeting P or vP criteria under specific environmental conditions (12 °C, pH 4–9), with water solubility and partition coefficients serving as key determinants of substance mobility (Neumann and Schliebner 2017). Toxicity is assessed based on criteria such as long-term no observable effect concentration (NOEC) or effect concentration at 10% (EC10) (Carlier et al. 2024; Arp and Hale 2022) for aquatic organisms, as well as classifications for carcinogenicity and reproductive toxicity. A substance is considered toxic if it meets any of these criteria, indicating potential harm to health or environment.

The EU Horizon 2020 funded ZeroPM project aims to establish an evidence-based framework to reduce persistent and mobile substance pollution. Within ZeroPM, a risk-based strategy was proposed as part of a tiered testing strategy to prioritize potential PMT or vPvM compounds (Gouin et al. 2024). This approach aims to strengthen decision-making processes regarding regulatory actions for these chemicals, which could include additional data acquisition to reduce uncertainties or implementing prevention measures to limit their exposure. The framework includes exposure and toxicity assessments, which integrate new approach methodologies (NAMs), such as in vitro bioassays and physiologically based kinetic (PBK) modelling (Gouin et al. 2024). The use of NAMs provides mechanistic insights into chemical modes of action, thereby enhancing our understanding of potential risks to human health and ecosystems while aligning with societal values on reducing animal testing (Colbourne et al. 2025).

Within the NAM-based approach, the PBK modelling is used to predict the internal human equivalent dosage of a compound based on its kinetic properties and external exposure. A PBK model represents the human organism as a set of compartments with mathematical equations connected by blood flow. The model is parameterized with compound-specific in vitro ADME data, hence referred to as an IVIVE bottom-up PBK model (Fabian et al. 2019; Bell et al. 2018; Linakis et al. 2025; Chang et al. 2022). Key ADME chemical specific parameters such as the free unbound fraction in plasma (*fub*), intrinsic hepatic clearance (*Clint*), blood-plasma ratio *(b/p)*, and intestinal permeability (*Papp*) are estimated using in vitro test systems. Additionally, physicochemical properties—including maximum solubility, the octanol-water partition coefficient (*logPow*), and Henry’s law constant for vapor pressure (*H pc*) are incorporated from established databases (Kim et al. 2025; Williams et al. 2017) to parameterize the model.

The bottom-up PBK model predicts the kinetic properties and bioavailable fraction of a compound, such as systemic clearance, C_max_ (maximum concentration in plasma), T_max_ (time at which C_max_ is attained), and AUC (area under the concentration-time curve).

The proposed modelling framework can only be applied to data-rich compounds. To address the challenges associated with the data-poor compounds, Escher et al. (2019) have proposed a read-across assessment framework that integrates New Approach Methodologies (NAM) data for kinetic and hazard assessment. This framework highlights the importance of read-across approaches in physiologically based kinetic (PBK) modelling when in vivo kinetic data is unavailable. PBK models for ‘source chemicals’ (data-rich chemicals) can effectively predict time-concentration dose metrics for ‘target chemicals’ (data-poor chemicals) that share similar absorption, distribution, metabolism, and excretion (ADME) properties (Rovida et al. 2021; Paini et al. 2021). The selection of appropriate analogues is crucial and can be based on structural, physicochemical, and ADME-related properties A structured workflow for identifying analogues and assessing their relevance to fill data gaps through read-across methodologies is provided, while also emphasizing the use of compound-specific in vitro ADME data to parameterize the validated PBK models for data-poor analogue compounds (Paini et al. 2021; Najjar et al. 2022).

Recent work on a set of 38 compounds demonstrated that relatively simple bottom-up PBK models can reasonably predict the bioavailable plasma concentrations (C_max_), within a factor of 10 when compared with the in vivo data (Cable et al. 2025). This work indicates that the use of IVIVE bottom-up PBK models for in vitro to in vivo extrapolation is conservative, as it tends to overestimate the bioavailability of compounds. The starting point of the QIVIVE are in vitro reference concentrations, which e.g. characterise biological activities leading to adverse outcomes. In vitro kinetic models are often used to estimate the corresponding effective concentrations considering a potential loss of the test compounds due to plastic, protein binding or evaporation (Proença et al. 2021; Heringa et al. 2004; Teeguarden and Barton 2004). The correction to effective concentration allows to compare and to integrate data from different in vitro models into the NAM based hazard assessment.

Most in vitro kinetic models assume instantaneous equilibrium using partition coefficients (Heringa et al. 2004; Kramer et al. 2009; Fischer et al. 2017; Fischer et al. 2019; Comenges et al. 2017), while some incorporate kinetic processes with rate constants or diffusion equations to capture time-dependent distribution (Stadnicka-Michalak et al. 2014; Comenges et al. 2017). The suitability of each model depends on the chemical’s properties (e.g., neutral vs. ionized, volatile vs. non-volatile) and the experimental setup (e.g., serum presence, plastic type, static vs. agitated systems). The VIVD model (Fisher et al. 2019) also accounts for the distribution within cells, including organelles like mitochondria and lysosomes. By extending its applicability to ionizable compounds—an advance over its predecessor (Armitage et al. 2014) the VIVD model is well-suited for estimating free, unbound concentrations in the medium for both ionizable and non-ionizable e.g. persistent and mobile compounds.

Several studies have successfully applied QIVIVE to extrapolate human equivalent doses and concentrations from in vitro test results. (Fabian et al. 2019) used reverse dosimetry to estimate human LOAELs for potential endocrine disruptors based on in vitro yeast assay data. Their results showed that for six out of ten chemicals, predicted LOAELs matched well in vivo values within the same order of magnitude, demonstrating the value and some limitations of PBK modelling in risk assessment.

Wetmore et al. (2012) integrated dosimetry, exposure, and high-throughput screening data for 239 chemicals. By including hepatic metabolic clearance and plasma protein binding information, the study improved predictions of human oral equivalent doses needed to reach in vitro bioactive concentrations (AC50 and lowest effect concentrations), highlighting the importance of kinetics in chemical prioritization.

Wambaugh et al. (2025) describe integrating NAMs into toxicokinetic modeling for data-poor chemicals, using QIVIVE to link in vitro data to in vivo outcomes and establish toxicological points of departure. An HTTK framework combines in vitro TK data with open-source models to rapidly screen many chemicals, with decision trees ensuring transparent, reproducible HTTK applications in safety assessments (Pearce et al. 2017). The research article from Honda et al. (2019) show PBK modelling for 84 chemicals strengthens the in vitro bioactivity–in vivo toxicity link (AC50), highlighting hepatic clearance and human internal bioavailable concentrations for more accurate predictions.

The study from Zobl et al. (2024) emphasizes the advancement of NAMs in toxicology, specifically focusing on the development of an in vitro test battery designed to derive protective points of departure (PoDs) for preclinical systemic toxicity. The article addresses the ethical concerns with the traditional animal-based methods and how NAMs play a role as an alternative to animal testing. The study shows for 5 chemicals that a high throughput in vitro test battery with human-based cell models can effectively predict human equivalent concentrations being lower and thus protective compared to those derived from traditional in vivo studies.

The advancements of NAMs approaches in toxicology over traditional animal methods are recognized increasingly for their human relevance and the ethical concerns that arise (Ouedraogo et al. 2025). NAMs tend to achieve higher throughput, leverage more relevant model systems, and be quicker and cheaper, with reduced reliance on animal testing. This combination can enhance the generation of safety data for most industrial chemicals (approximately 70%) that have not been fully evaluated for potential human adverse effects (Browne et al. 2024).

The present study develops a QIVIVE approach for two classes of persistent and mobile pesticides, specifically triazoles and triazines. These two chemical classes were selected as model compounds because many of these pesticides possess extensive preclinical in vivo ADME and hazard datasets. This enables a direct assessment of the predictivity of the NAM-based approach in comparison to traditional in vivo animal data. Similar QIVIVE approach with ToxCast data (2015) was performed in the following studies by Loizou et al. (2021) and Loizou et al. (2022) for PFOA and bisphenols.

The in vitro bioactivity data were taken from Carlier et al. (2024), who determined in vitro bioactivity profiles for 36 persistent and mobile compounds, including 9 triazines, 16 triazoles, and 11 short-chain poly- and per- fluoroalkyl compounds (PFAS). The study employed a battery of NAM assays associated to endocrine activity, evaluating the agonistic and antagonistic effects on estrogen receptors (ER), androgen receptors (AR), thyroid hormone receptors (TR) using reporter gene assays along with H295R steroidogenesis.

Two bottom-up PBK models were built per class following the recently developed NAM based read-across assessment concept of the EUTOXRISK project (Escher et al. 2019). The PBK models used the structure of the Fraunhofer PBKiT model (Nowak et al. 2025; Spaenig et al. 2025) and applied in vitro measured compound-specific ADME data, including a permeability estimated from a Caco-2 barrier model (*Papp*), the fraction unbound (*fub*), as well as intrinsic metabolic clearance values estimated from primary human hepatocytes and/or microsomes (*Clint*).

In our study, the comparison of the measured in vivo plasma concentration to predicted plasma concentration in rat indicated a good predictivity of the bioavailable concentration in plasma as per OECD (2021) guidance document. The validated PBK model’s approaches were translated to a human PBK model and used for the other compounds in the triazine/triazole classes. Sensitive in vitro ADME input parameters for data poor compounds were predicted using the read-across approach or a worst-case approach.

An in vitro to in vivo extrapolation was performed by first correcting the reference in vitro benchmark concentrations (e.g. EC20, IR1.5) to the corresponding free effective unbound medium concentrations. The resulting human equivalent plasma concentrations were compared to reference values from in vivo toxicity studies as well as surface and groundwater levels to assess the margin of safety resulting from the of the QIVIVE approach.

## Materials and methods

### Compound selection

Two classes of persistent and mobile compounds were selected namely triazoles and triazines. **Triazoles** are a class of persistent and mobile (PM) compounds, featuring a five-membered aromatic ring structure that includes three nitrogen heteroatoms. These compounds are primarily used as corrosion inhibitors (Hrimla et al. 2021), fungicides and as plant growth retardants (Jónsdóttir et al. 2016). A total of 16 triazoles were selected, of which four—tebuconazole (Jónsdóttir et al. 2016; Oerlemans et al. 2019; EFSA DAR (2007) - Tebuconazole), triadimenol (Crowell et al. 2011; EFSA DAR (2006) – Triadimenol), 1,2,4-triazole, and triazole acetic acid—are classified as data-rich (Kim et al. 2025; Williams et al. 2017; ACD/Labs 2023), while the remaining 12 compounds are considered data-poor (benzotriazole, 4-methylbenzotriazole, 5-methylbenzotriazole, triazole alanine, cyproconazole, tetraconazole, propiconazole, difenoconazole, fenbuconazole, bitertanol, and pyroxsulam. (Supplementary Sect. 1).

**Triazines** are another class of persistent and mobile (PM) compounds characterized by a six-membered aromatic ring containing three nitrogen heteroatoms. These compounds are primarily utilized as fungicides (Henriquez et al. 2023), as herbicides (LeBaron et al. 2008) and as components of resins for example melamine (Bui et al. 2020). A total of nine triazines were selected for this study. Among them, four compounds—cyromazine (EFSA DAR (2007)- Cyromazine), melamine (National Toxicology Program 1983 – Melamine; Buur et al. 2008), atrazine (Campbell et al. 2016; McMullin et al. 2007), and ammelide - are considered data-rich (Kim et al. 2025; Williams et al. 2017; ACD/Labs (2023), as they possess in vivo hazard data on systemic and developmental toxicity with preclinical LOAELs. The remaining five compounds, classified as data-poor, include cyanuric acid, deethylatrazine, ammeline, benzoguanamine, and ametryn (Supplementary Sect. 1).

### Bottom-up PBK modelling approach

**Fig. 1:**
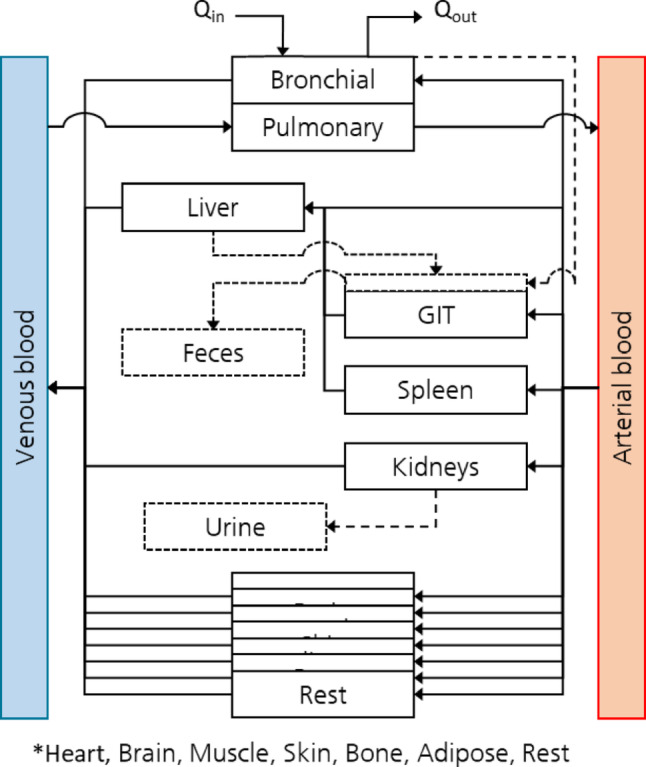
Structural presentation of the Fraunhofer ITEM developed bottom-up PBK Model (PBKiT) (Nowak et al. 2025; Spaenig et al. 2025). The model can address the typical administration routes of exposure such as, (1) oral (2) inhalation or (3) dermal uptake for the compounds

The PBKiT model (Fig. 1) (Fraunhofer ITEM) is designed to serve as a generic framework which can be parameterized with chemical specific NAM derived ADME data. The model possesses well established skin, lungs, liver, kidney and gastrointestinal tract (GIT) compartments. The model possess brain, muscles, skeleton, heart, adipose tissues (fat), spleen as the additional physiological compartments. The rest compartment acts as the reservoir to collect the excretion from the kidney output.

The rat model is designed to handle gavage and oral administration (food or drinking water). The following physiological parameters are considered in the rat gastro - intestinal tract (GIT) (Table 1). The pH difference between the fasted and the fed state is an integral part of the absorption in the oral route amongst the different sub compartments (duodenum, jejunum, ileum, colon and rectum) of rat GIT. To parametrize the difference in volumes in the fasted and fed state along with these pH differences, the measured values from McConnell et al. (2008) are used.

To construct the bottom-up rat PBK model, the volumes of stomach and forestomach has been merged into a single sub-compartment, as rat’s forestomach acts as a reservoir for storage without involving the actual digestion process (Gärtner 2002). The gastric secretion and emptying are adopted from Paré (1977) and Kaye (1971). The overall clearance from the rat body occurs through a combination of two pathways: the intrinsic hepatic clearance, which follows the well-stirred model (Pang et al. 2024) mechanism similar to humans (Kaye 1971; Pang and Rowland 1977) and the renal elimination via glomerular filtration rate (GFR). The value of GFR equalling 9.6 ± 0.6 mL/min/kg of a healthy male F344 rat is adopted from Katayama et al. (2010).

**Table 1:** Physiological parameters – Sprague Dawley rat and human male

Parameters		Unit	Rat	Human male ^i^
Body weight		Kg	0.35 ^a^	70
Blood volume		L	0.02 ^b^	5.30
Volume (fasted)	Stomach	mL	3.40 ^c^	47
Length	Small intestine	cm	148.50 ^d^	700
	Large intestine	cm	26 ^d^	110
Diameter	Small intestine	cm	0.25–0.50 ^d^	4–2.40
	Large intestine	cm	1–0.30 ^d^	6−4
Surface Area	Small intestine	cm^2^	67.88 ^d^	2E+6
	Large intestine	cm^2^	27.06 ^d^	3460
pH (Fasted and Fed)	Stomach	No units	3.90 ^c^	1.90
	Small intestine	No units	5.89–6.13 ^c^	6.12–7.42
	Large intestine	No units	5.64–6 ^c^	6.30–7
GIT transit	Stomach	h	0.50 ^e, f^	0.50
	Small intestine	h	4 ^e, f^	4
	Large intestine	h	12 ^e, f^	40
Gastric secretion (fasted)	Stomach	mL/h	1.50 ^h^	133.33
	Small intestine	mL/h	3.80 ^h^	164.58
	Large intestine	mL/h	0*	2.50

*Sources:* The human physiological parameters are adopted from the rat physiological parameters are from the following sources:

^a^ Cao et al. (2020)

^b^ Probst et al. (2006)

^c^ McConnell et al. (2008)

^d^ Vdoviaková et al. (2016)

^e^ Dalziel et al. (2016)

^f^ Purdon and Bass (1973)

^g^ Paré et al. (1977)

^h^ Ruderman and Nagy ( 1974)

^i^ Spaenig et al. (2025)

* Assumption.

1 $${\text{Primary Human Hepatocytes }}\left( {{\mathrm{PHH}}} \right) \to Clint_{{Scaled}} = \frac{{CL_{{int}} }}{{fub}}*HPGL*Wl$$

2 $${\mathrm{Microsomes}} \to Clint_{{Scaled}} = \frac{{CL_{{int}} }}{{fub}}*MPPGL*Wl$$

Scaling up the in vitro hepatic clearance to the rat and human liver for the respective models is carried out by ‘Eq. 1’ and ‘Eq. 2’ (Musther et al. 2017) in case of the primary human hepatocytes (PHH) or microsomes. *Clint* represents the in vitro hepatic clearance from primary human hepatocytes as µL/min/10^6 cells or as µL/min/g proteins. *Clint*_*scaled*_ represents the up scaled *Clint* in the entire liver either in rat or human. *Wl* represents the liver weight (g), which is estimated to be 11.9 g for a 350 g male rat (ICRP 2002). *fub* represents the protein plasma fraction unbound. *MPPGL* represents human microsomal protein per gram of liver and *HPGL* represents the hypocellularity per gram of liver. The renal clearance is calculated by the product of free fraction unbound (Eq. 3) of the substance to the protein plasma (*fub*) and the glomerular filtration rate (GFR) (Cristea et al. 2020).

3 $${\text{Renal clearance}} = {\text{GFR }}*fub$$

**Table 2:** Physiological parameters with regards to metabolism and excretion for the rat and human bottom-up PBK model

Parameter	Value – Rat (350 g)	Value – Human (70 kg)	Unit
Weight of liver ^a^	11.90	1800	g
HPGL ^b^	107E+6	117.50E+6	Cells/g liver
MPPGL ^b^	46	40	mg/g liver
Glomerular filtration rate (GFR)	9.60 ^c^	125 ^d^	mL/min/kg

^a^ ICRP (2002)

^b^ Musther et al. (2017)

^c^ Katyama et al. (2010)

^d^ Levey et al. (2014)

The key distinction between the oral routes in rats and humans lies in the anatomical structure and different physiological parameters (Tables 1 and 2). In the rat model, the forestomach and stomach are combined into a single compartment, while humans do not have a forestomach.

Partition coefficients for the persistent and mobile (PM) compounds were modelled with the Rodgers and Rowland (Rodgers et al. 2005; Rodgers and Rowland (2006) method for the rat and human models. The in vitro measured ADME parameters such as intrinsic hepatic clearance *(Clint)*, fraction unbound *(fub)*, blood - plasma ratio *(b/p)* and the apparent permeability *(Papp)* which were measured using human cell lines were also used for bottom-up rat modelling, with the respective scale up factors.

### Permeability measurements from Caco-2 assay

Caco-2 cells (clone C2BBe1, ATCC-HTB-37) to measure the intestinal permeability were obtained from LGC Standards GmbH (Wesel, Germany) and cultured in DMEM high glucose medium supplemented with 10% FCS, 1% non-essential amino acids, 1% sodium pyruvate, 10 µg/ml transferrin, and 0.01% gentamycin. The cells were seeded in 12-well inserts at a density of 5 × 10^4^ cells per well and cultured for 10–15 days to form a tight monolayer with a transepithelial electrical resistance (TEER) greater than 500 Ωcm^2^, indicating proper differentiation. For intestinal barrier transport experiments, two test concentrations (1 µM and 10 µM) were used, and the apical compartment was dosed with 500 µL medium containing the test substances. During the measurement, three biological replicates were established, and samples of the basolateral medium were collected at 30, 60, 90, and 120 min. The apical medium and cell fractions were harvested at the 120-minute mark. All permeability experiments were conducted under sink conditions, with the receiver compartment being exchanged at each sampling interval (Artursson and Karlsson 1991; Artursson and Borchardt 1997; van Breemen and Li 2005).

4 $$\:{P}_{app}={V}_{r}*\left(dC\right)/\:\left(dt\right)*(1/\left(A*{C}_{o}\right))\:$$

Where,

*Papp*: Apparent permeability. (cm/s)

*Vr*: Volume of the recipient compartment.

*dC/dt*: slope of the cumulative concentration of the compound in the recipient chamber over time (nmol/s).

*A*: area of transwell (cm^2^).

*C*_*0*_: initial concentration of product applied in the apical (A-B) or basal (B-A) compartment (nmol/ml).

### Intrinsic hepatic clearance

The in vitro human hepatic metabolic clearance method involves exposing cryopreserved pooled primary human hepatocytes to a final concentration of 1.5 µM of the compound being tested. The cell incubation concentration is set at 0.525 × 10^6 viable cells/mL in an incubation volume of 100 µL. Following thawing, cell viability was assessed at 91.6%. The incubation times are designated at 0, 0.25, 0.5, 1, 2, and 4 h, with samples of three biological replicates placed in rows 1–6 of the 96-well plate. At the end of each time point, the well contents consist of 100 µL of the incubation aliquot mixed with 100 µL of acetonitrile, which contains 0.1 µM warfarin and 0.2 µM carbutamide as the internal standards.

The calculated concentrations from the measured triplets, expressed in nM, were transformed to a natural logarithmic scale (ln) for analysis. The elimination rate constant (k) was determined from the slope of ln (Ct / C0) versus time (t, in minutes), using a 1/y weighting, as per ‘Eq. 5’:

5 $$ln(C_t/C_0) = - \,kt$$

where *C*_*t*_ is the substrate concentration at time t and *C*_*o*_​ is the initial substrate concentration at *t* = 0. The intrinsic clearance in vitro *(Clint)* was then calculated using the formula:

6 $$\:Cl_{{int}} = k*\frac{V}{N}$$

{μL/min * 10^6 cells}.

with *k* equal to 0.639 / t_1/2_​, *V* as the incubation volume (100 µL), and *N* representing the number viable cells used in this assay.

### Plasma protein binding

Pooled human plasma was provided by Uppsala Academic Hospital and collected from two non-smoking donors using citric acid as an anticoagulant.

Briefly, 0.2 mL of the plasma test solution (50% plasma, 50% isotonic buffer), typically containing a final compound concentration of 10 µM, was transferred to the membrane tube in the Rapid Equilibrium Dialysis (RED) insert (Thermo Fisher Scientific). To the opposite side of the membrane, 0.35 mL of isotonic phosphate buffer (pH 7.4) was added.

The 96-well base plate was sealed with an adhesive plastic film (Scotch Pad) to prevent evaporation. Samples were incubated at 37 °C with rapid rotation (approximately 900 rpm) on a Kisker rotational incubator for 4 h to reach equilibrium. To assess compound stability, approximately 100 µL of the plasma test solution (in a plastic vial or on a sealed plate) was incubated at 37 °C for 4 h (equal to the dialysis time). Plasma test solutions were frozen immediately after preparation to prevent degradation. Prior to LC-MS/MS analysis, both plasma and buffer samples were treated with methanol (1:3 ratio) containing warfarin as an internal standard to precipitate proteins. A standard curve was generated using plasma standards.

Finally, the plate was sealed, centrifuged, and the supernatant was analysed by liquid chromatography coupled to triple quadrupole mass spectrometry (LC-MS/MS). This same method is repeated for all three biological replicates. The fraction drug bound (*fub*) is determined using the relationship in ‘Eq. 7’ (Mamada et al. 2021), where buffer concentration (*C*_*buffer*_) and plasma concentration (*C*_*plasma*_) are the concentrations obtained by LC-MS/MS analysis of the plasma and buffer samples, respectively.

7 $$\:{f}_{ub}=\frac{{C}_{unbound}}{{C}_{total}}=\frac{{C}_{buffer}}{{C}_{plasma}}$$

### Blood – plasma ratio

In the blood to plasma ratio assay, human whole blood from two donors was pooled, with a measured haematocrit of 0.375. The compounds were incubated at a final concentration of 10 µM with either whole blood or plasma at room temperature. Samples were collected at 15, 30, and 60-minute intervals. For whole blood, aliquots were centrifuged at 5000 x g for 5 min, and 50 µl of plasma was precipitated in 150 µl acetonitrile. Plasma samples were processed using the same time points. After centrifugation, proteins were removed, and 100 µl of the supernatant was transferred to a new 96-well plate for analysis. The plate layout included rows for whole blood and plasma incubations at each time point. Internal standards were used to ensure consistent analysis across wells, accounting for potential variations in volume. Three biological replicates were put in place during the measurement to achieve statistical significance. The following equation (Eq. 8) to calculate the blood – plasma ratio is from Uchimura et al. (2010).

8 $$\:BP\:Ratio\:\left(\frac{{K}_{b}}{p}\right)=\left(\frac{{K}_{e}}{p}*H\right)+\left(1-H\right)$$

*K*_*b*_ /* p* → Blood plasma ratio

*H* → Haematocrit

*K*_*e*_ /* p* → Erythrocyte plasma ratio

### LC – MS analysis

LC-ESI-MS/MS coupling was used to analyse 1,2,4-triazole, ammelide, triazole acetic acid, triadimenol, tebuconazole, cyromazine, and melamine in the samples. Chromatography was performed using an Agilent 1290 Infinity II system and, depending on the analyte group, a Restek Ultra Aromax, Halo RP-Amide or Thermo HyperCarb column. Detection was performed using a Sciex QTrap 6500+ mass spectrometer. Evaluation and data processing was performed using Sciex OS 4.0 software.

### Sensitivity analysis

Morris method of sensitivity analysis was applied to evaluate the influence of compound specific ADME input parameters on model outputs (Covington and Gearhart (2020)). This technique involves systematically perturbing input parameters to assess their impact on the model’s outputs. The sensitivity coefficient (SC) quantifies this effect using 'Eq. 9':

9 $$\:SC=\frac{f\left({\theta\:}_{1}\right)-\:f\left(\theta\:\right)}{f\left(\theta\:\right)}*\frac{{\theta\:}_{1}-\:\theta\:}{\theta\:}$$

where *f(*θ*)* represents the model output with the original parameter value θ, and *f*(θ1) is the output after perturbation. Morris method highlights the need for multiple changes across a two-fold space to create a full sensitivity profile, helping identify key model parameters.

Additionally, Markov Chain Monte Carlo (MCMC) simulation was employed as an advanced method that builds on traditional Monte Carlo techniques by using prior distributions and experimental data to estimate uncertainty in model parameters (Spaenig et al. 2025; Covington and Gearhart 2020). MCMC updates the probability distribution of parameters based on new data, improving predictions. The process utilized a two-fold range to determine uncertainty, facilitating the creation of posterior distributions that reflect updated knowledge after each step, based on:

10 $$\:P\left(\theta\:|D\right)\:\propto\:P\left(D|\theta\:\right)*P\left(\theta\:\right)$$

where P(θ∣D) is the posterior distribution, P(D∣θ) is the likelihood of the data given the parameters, and P(θ) is the prior distribution. MCMC simulations are particularly valuable in PBK modelling for quantifying uncertainties in parameter estimates and enabling more accurate predictions of pharmacokinetic behaviour across populations.

### In vitro biokinetic modelling and QIVIVE approach

The free medium in vitro benchmark concentrations per in vitro assay was calculated using the VIVD model (Fisher et al. 2019). The VIVD model considers the interaction of the substance with the cellular system like its partitioning to air, cells, proteins suspended in the medium and to the plastic surface area in contact to the medium. Input parameters per assays system are listed in the Supplementary Sect. 2. For ionizable compounds the VIVD model uses the pKa, compound type (neutral, acidic, basic, monoprotic acid, monoprotic base, zwitterions) and the corresponding logPow parameters to account for the adopted partition coefficients to the different compartments within the cell system (Supplementary Sect. 2).

‘Table 3’ shows the physicochemical parameters needed as an input to the VIVD model to perform the in vitro correction (Supplementary Sects. 2, 3). The corrected free medium in vitro benchmark concentration for the measured assays for tebuconazole from Carlier et al. (2024) is included as a part of ‘Table 4’ (also Supplementary Sects. 3 and 5). Further the complete set of input parameters per assay and compound as well as the correction factor per assay and compound are listed in the Supplementary Sect. 2.

**Table 3:** VIVD model input parameters

Compound name	Tebuconazole	Cyromazine
Fraction unionized	1	0.99
Henry’s partition coefficient *(H pc)* (Pa*m^3^/mol)	3.14E-02	9.53E-4
Compound Type at pH 7.4	Neutral	Monoprotic acid
pKa1	1.75	5.30
pKa2	13.68	Not available
logPow	3.73	− 1.22
Intrinsic hepatic clearance (*Clint)* (µL/min/10^6 cells)	5.28	0.04
Molecular weight (g/mol)	307	166.09

* Complete set of input parameters for all selected compounds are included in the Supplementary Sect. 2

**Table 4:** VIVD model correction – Tebuconazole correction (concentration in mol/L)

Assays	H295R Steroidogenesis (progesterone)	Androgen receptor assay antagonism (AR)	Estrogen receptor assay antagonism (ER)
Nominal concentration (EC20, IR1.5) reported by Carlier et al. (2024)	4.14E-6	2.99E-6	2.44E-5
Free medium in vitro benchmark concentration (free EC20, IR1.5)	1.39E-6	6.14E-7	4.98E-6
Protein concentration	2.52E-6	2.23E-6	1.81E-5
Concentration in air	4.48E-11	1.99E-11	1.61E-10
Concentration partitioned to plastic	4.47E-7	1.98E-7	1.60E-6
Concentration partitioned within cells	1.46E-4	6.46E-5	5.23E-2

* All the corrected free medium concentrations for all assay types from Carlier et al. (2024) is included in the Supplementary Sects. 3 and 5

‘Table 4’ gives the effective concentration in the form of ‘free medium in vitro benchmark concentrations’ from the nominal concentrations (EC20 and IR 1.5). Along with these values, the predicted partitioned concentrations into protein in the system, air, plastic and within the cells are included in the table.

### Exposure scenario for triazoles and triazines

The exposure scenario used in this study considers a 70 kg human male exposed to triazole or triazine compounds through consumption of 2 L of contaminated water per day. Dosing occurs at 0, 4, 8, 12, and 16 h daily, followed by an 8-hour overnight break, with this pattern repeated for 5 consecutive days (Table 5).

11 $$\begin{aligned} Total~Exposure{\text{ }} = & {\text{ }}Dissolved{\text{ }}chemical{\text{ }}concentration{\text{ }}in{\text{ }}water{\text{ }} \\ & \times {\text{ }}2\,L{\text{ }}/{\text{ }}day{\text{ }} \times {\text{ }}5\,days \\ \end{aligned}$$

**Table 5:** Drinking water oral route exposed concentrations and the corresponding PBK model derived free unbound blood - plasma concentrations for model compounds Tebuconazole and cyromazine

Source of concentration	Tebuconazole -total exposure	Exposure units	Tebuconazole – free unbound C_max_ (mol/L)	Cyromazine - total exposure	Exposure units	Cyromazine -free unbound C_max_ (mol/L)
Allometrically scaled LOAEL (EFSA) ^a, b^	1.37	mg/kg bw	3.28E-5	9.90 mg/kg bw	mg/kg bw	1.048E-4
Ground water concentration ^c^	6.65E-4	µg/L	2.21E-13	0.07 µg/L	µg/L	5.36E-12
Surface water concentration ^c^	65	µg/L	5.34E-9	21 µg/L	µg/L	3.20E-9
Maximum water solubility (PubChem) ^d^	3.60E+4	µg/L	2.98E-6	1.30E+7 µg/L	µg/L	1.98E-3

* Complete list of exposed ground water, surface water and LOAEL input concentrations and the corresponding C_max_ is depicted in ‘Supplementary Sect. 4’.

^a^ EFSA DAR (2007) - Tebuconazole

^b^ EFSA DAR (2007)- Cyromazine

^c^ EMPODAT Norman Database (2020)

^d^ Kim et al. (2025)

### Read – across approach to estimate in vitro ADME data

The read-across approach estimates missing in vitro ADME parameters for data-poor compounds based on physicochemical properties and chemical structures. This method derives key values including apparent permeability (*Papp* in cm/s) for absorption, plasma protein fraction unbound (*fub*) for distribution, and intrinsic hepatic clearance (*Clint*) for metabolism for the data – poor compounds. A detailed list of compounds and corresponding ADME parameters used for read-across is provided in Supplementary Sect. 1.

For intrinsic hepatic clearance (*Clint*), compounds lacking the in vitro measured *Clint* values the worst-case assumption of no clearance (*Clint* = 0) is included in the modelling process, as all the PM compounds are generally characterized as low clearance compounds (Esaki et al. 2019). Missing fraction unbound (*fub*) values, which were not obtained from in vitro measured data or the CompTox dashboard (Williams et al. 2017) are supplemented with values from the OPERA open-source software (Mansouri et al. 2018).

Regarding the blood - plasma (*b/p*) ratio, a value of 1 is assigned to all data-poor compounds. This choice is based on the observation that the *b/p* ratios for measured compounds are consistently close to 1. The apparent permeability value for a group of compounds (triazines or triazoles) (*Papp*) is derived from the average of other measured values, as outlined in the ‘Supplementary Sect. 1’.

## Results

### Rat PBK model

A bottom-up PBK model incorporating physicochemical properties and compound-specific in vitro ADME data was used to predict in vivo rat pharmacokinetics for tebuconazole and cyromazine. The ADME data were derived from human in vitro models, assuming cross-species similarities between humans and rats, such that each parameter is predicted to fall within the same range across the test compounds (e.g., high, moderate, or low clearance). This assumption cannot be proven for all compounds in this paper, but it is supported by data for one representative of each compound class—for example, tebuconazole (Jónsdóttir et al. 2016; Oerlemans et al. 2019) and atrazine (Campbell et al. 2016)—which showed comparable in vivo ADME parameters in rodents and humans.

**Table 6:** Compound specific physicochemical and in vitro measured ADME data for representative compounds per class – tebuconazole and cyromazine

Parameters	Tebuconazole	Cyromazine
logPow	3.73	0.49
Water Solubility (mg/L)	36 ^a^	1.30E+4 ^a^
Fraction Unbound (*fub*)	0.06 +/− 0.001	0.51 +/− 0.13, 0.94 ^b^
*Papp* * 1E-6 (cm/s)	3.92+/− 8.85E-1	2.90E+/− 1.30E-1
*b/p* ratio	0.94 +/− 0.21	0.90 +/− 0.09
Fraction absorbed by the intestine (h^− 1^)	1.40E-2	1.04E-2
*Clint* PHH (µL/min/10^6 cells)	3.62+/− 0.02, 5.27 +/− 0.01^b^, 7.30 +/− 0.13*	0.90 ^b^, 0
*Clint* PHH scaled to rat (mL/h)	6897.65 10060.66 13909.63	235.37
*Clint* PHH scaled to human (mL/h)	716760 1043460 1445400	20964.71
*Clint* Microsomes (µL/min/g protein)	53 ^c^	–
*Clint* Microsomes scaled to rat (mL/h)	397.05	–
*Clint* Microsomes scaled to human (mL/h)	381600	–

*Data provided by the RISK-HUNT3R project

^a^ Kim et al. 2025

^b^ Williams et al. 2017

^c^ Habenschus et al. 2019

### Application of the rat PBK model to Tebuconazole

Tebuconazole was administered in the rat oral route PBK model at a single dose of 2 mg/kg body weight via gavage, consistent with the radiolabelled in vivo rat kinetics study outlined in the EFSA Draft Assessment Report (DAR) - Tebuconazole (EFSA DAR (2007) - Tebuconazole).

We compared the outcomes from primary human hepatocytes (PHHs) and human microsomes to evaluate the applicability of two different in vitro models for assessing intrinsic hepatic clearance. Pooled cryopreserved PHHs from 10 donors provided three comparable clearance values, ranging from 3.62 to 7.30 µL/min/10^6 cells (Table 6). The median clearance value of 5.27 µL/min/10^6 cells (Table 6) was scaled to total liver clearance by assuming an average rat liver weight of 19 g and a liver cell density of 107 cells/g for a typical 350 g male Sprague Dawley rat (Musther et al. 2017).

In comparison, human microsomes exhibited a clearance value of 53 µL/min/g protein, which was scaled to rat liver clearance using a total microsomal protein amount of 40 mg per gram of liver and again a total liver weight of 19 g (Musther et al. 2017) (Table 2, Eq. 2). The scaled intrinsic clearance *(Clint)* values indicate that the clearance of Tebuconazole from microsomes is three to four times higher compared to the clearance estimated from the PHH assays (Table 6).

The *Clint* values, along with the *fub* of the substance being as low as 0.06, indicate that the scaling of *Clint* from the in vitro measurements using PHHs to the whole liver is highly dependent on the *fub*. Equations 1 and 2 illustrate that the scaled intrinsic clearance value (*Clint*_*scaled*_​) is inversely proportional to the fraction unbound *(fub)*. This means that as the fraction unbound decreases (indicating more drug is bound to plasma proteins), the scaled intrinsic clearance also decreases. Consequently, the low *fub* of 6% suggests that Tebuconazole is not effectively cleared from the liver, reinforcing its classification as a low clearance compound (Esaki et al. 2019).

**Fig. 2:**
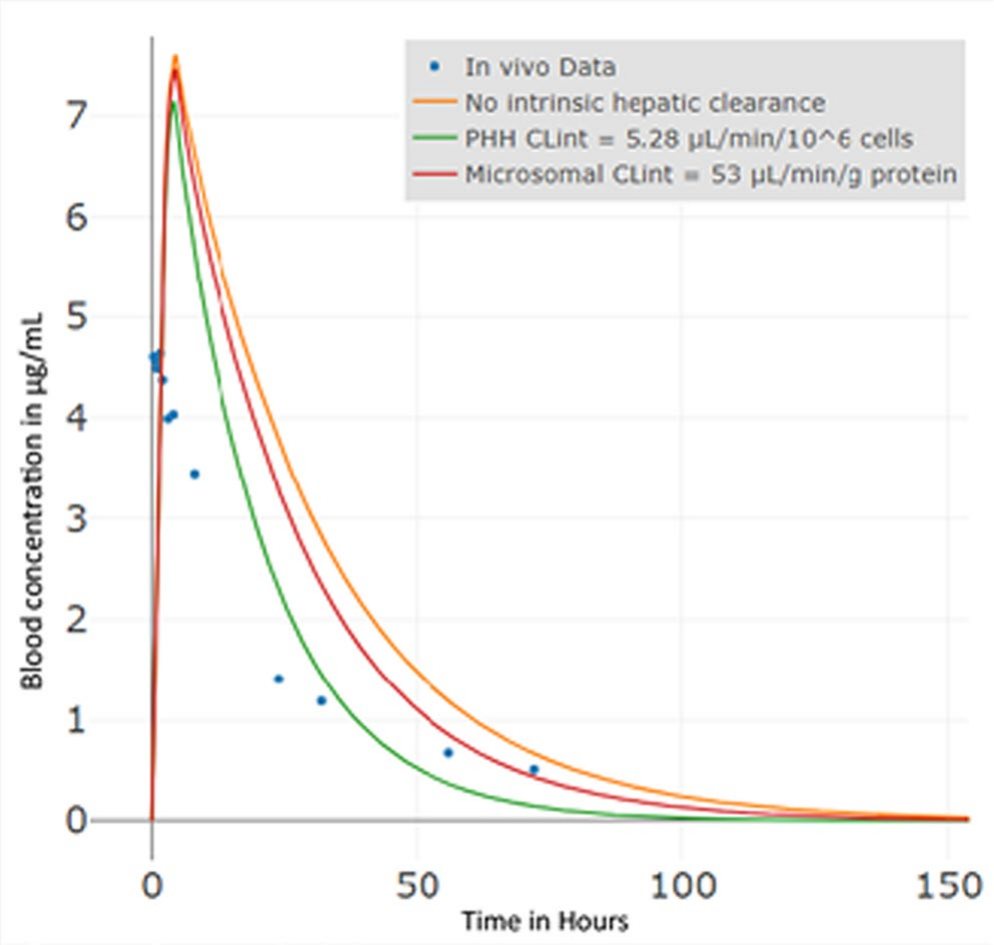
Bottom-up PBK model simulated plasma concentration–time profiles for tebuconazole in rats after a single oral dose of 2 mg/kg bw, evaluated under different hepatic clearance scenarios

The measured blood-to-plasma (*b/p*) ratio is close to 1 (0.94 +/− SD = 0.21), suggesting very less accumulation of tebuconazole in blood cells. he EFSA in vivo dataset (EFSA DAR (2007) - Tebuconazole) reports total radioactivity (parent plus metabolites) and is used as the reference profile for IVIVE–PBK evaluation. Despite this uncertainty, the observed blood – plasma concentration–time curve (Fig. 2) provides a suitable benchmark: given the low metabolic clearance of tebuconazole (Table 6), model predictions for C_max_ and T_max_ are within about two-fold of the in vivo values as per the OECD (2021) guidance document (Table 7). This aligns with Jónsdóttir et al. (2016), who showed that a simplified rat oral PBK model can reproduce the tebuconazole blood – plasma profile within roughly two-fold of observations.

**Table 7:** Tebuconazole PBK model results

Data type	PBK Model prediction			In vivo data (EFSA DAR (2007) - Tebuconazole)
	No hepatic clearance (factor)	PHH clearance (factor)	Microsomes clearance (factor)	
C_max_ (µg/ml)	7.61 (1.6)	7.14 (1.5)	7.47 (1.6)	4.63
T_max_ (h)	4.39 (2.8)	4.16 (2.7)	4.32 (2.8)	1.53
AUC (µg/mL * h)	232 (2.0)	143 (1.3)	198 (1.8)	112.36

A factor (indicated in brackets) is calculated as the ratio of model predictions to the measured in vivo data to assess the PBK model’s accuracy and performance through goodness of fit

The curve with ‘no hepatic clearance’ indicates only clearance by glomerular filtration rate (GFR). The model including PHH clearance data yields the best results as compared to the in vivo data (EFSA DAR (2007) - Tebuconazole). While the maximum concentration obtained in plasma (C_max_) values are similar across all three scenarios. Across the PBK scenarios, the model with PHH clearance fits the in vivo data best; microsomal clearance is second; the no hepatic clearance case is worst. C_max_ is overpredicted in all models, with PHH showing the smallest deviation (factor 1.5). T_max_ is consistently longer in the models. AUC follows the same pattern. Based on this, we extrapolate the PHH-clearance model as the best fit to the human model.

### Sensitivity analysis

The sensitivity analysis with Morris plot followed by the uncertainty analysis with MCMC analysis was performed for the compound specific ADME parameters namely intrinsic hepatic clearance (*Clint*), fraction unbound (*fub*), apparent permeability (*Papp*) and maximum water solubility of the chemical at 20 °C.

**Fig. 3:**
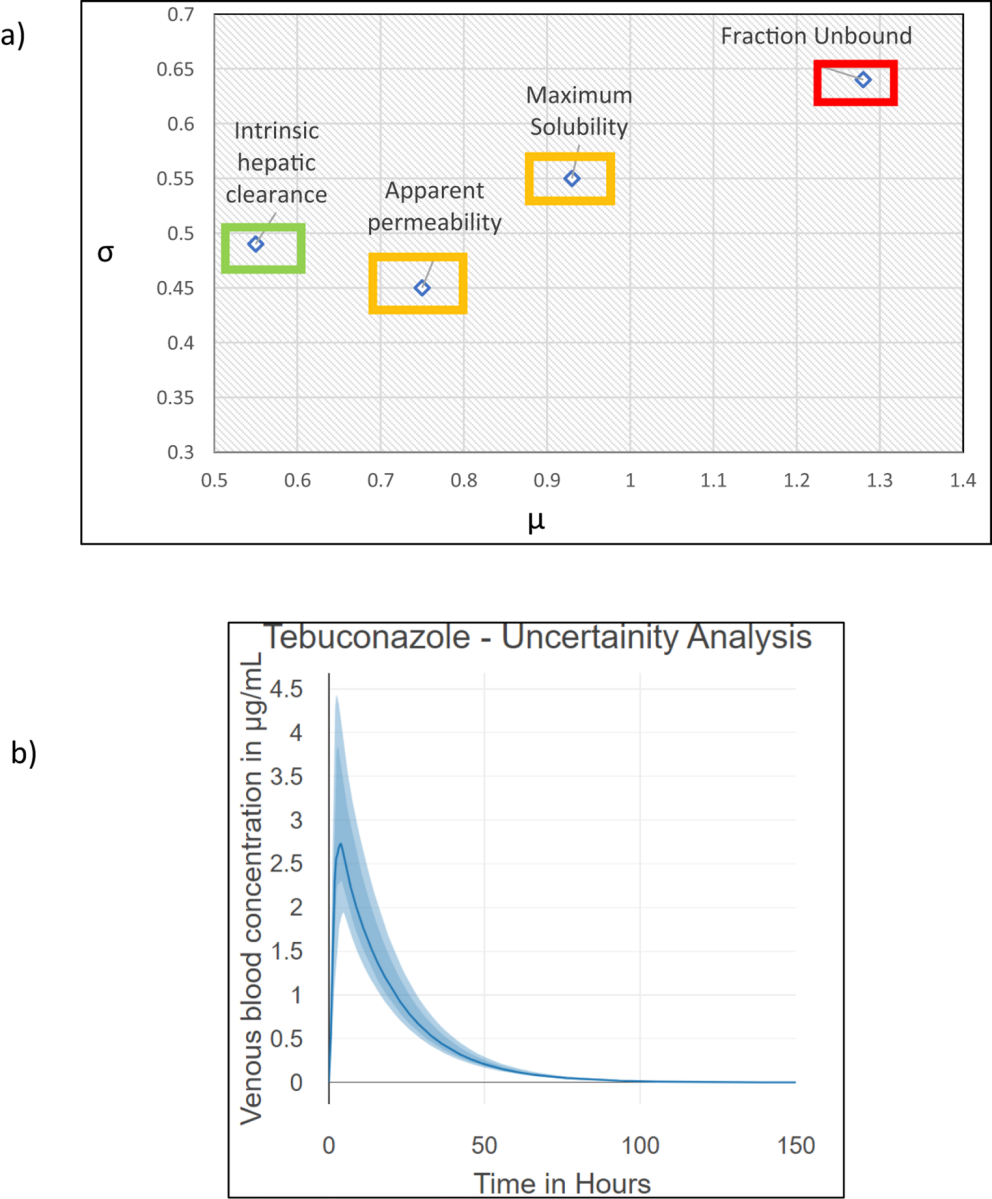
Tebuconazole** a** Sensitivity analysis by Morris plot** b** Uncertainty analysis with compound specific parameters within 2-fold range

For the sensitivity analysis the intrinsic hepatic clearance of 5.27 +/− 0.01 µL/min/10^6 cells were used as it showed the best fit in Fig. 2; Table 7. Figure 3a presents a Morris global sensitivity plot for the model outputs. The x-axis (µ) is the mean of the elementary effects and reflects each ADME parameter’s overall influence on the outcome, while the y-axis (σ) is the standard deviation of the elementary effects and captures nonlinearity and interactions among all four ADME parameters.

The highest sensitive parameters in decreasing order are fraction unbound > maximum solubility > apparent permeability > intrinsic hepatic clearance. The sensitivity analysis indicates that the fraction unbound creates the highest impact for modelling tebuconazole as the *fub* value is very small as 0.06 (Table 6), constituting a big difference with respect to the bioavailability when provided with 2-fold sample space. On contrary, the intrinsic hepatic clearance does not show a major impact as in general, the triazoles have low intrinsic hepatic clearance values *(Clint)*.

Figure 3b shows the MCMC-based uncertainty for venous blood concentration over time. The solid line is the posterior mean; the darker band is the central credible interval, and the lighter band is a wider credible interval. Uncertainty is highest near the absorption peak (around C_max_) and steadily decreases as elimination proceeds.

### Cyromazine (Triazine) modelling and validation

The rat oral route PBK model was administered cyromazine (neutral at pH 7.4) at a single dose of 3 mg/kg body weight in poly-ethylene glycol used as a vehicle, as derived from the EFSA Draft Assessment Report (EFSA DAR (2007)- Cyromazine). The in vitro models for assessing metabolic clearance indicated that cyromazine exhibits very low metabolic clearance, with a *Clint* value of 0.9 µL/min/10^6 cells (Table 6). This low clearance is in accordance with published data from the CompTox dashboard (Williams et al. 2017; Esaki et al. 2019), which also reports an experimental PHH clearance value of 0 (Table 6).

The apparent permeability (*Papp*) of cyromazine is measured at 2.92E-6 cm/s, indicating low to moderate absorption level (Kus et al. 2023; Pham-The et al. 2013). The fraction unbound of the plasma protein (*fub*) is measured to be 0.51, while the CompTox dashboard (Williams et al. 2017) provided a higher value of 0.94 (Table 6). The *b/p* ratio of 0.90 is used for modelling (Table 6). These parameters were utilized as inputs for the rat oral route model to predict the biokinetic properties.

For cyromazine, the predominant pathway for elimination from the body is through glomerular filtration (Eq. 3) (Cristea et al. 2020), given the extremely low intrinsic hepatic clearance. The maximum water solubility of cyromazine at 20 °C, which reflects the highest concentration that could potentially be found in a drinking water scenario, is reported as 13 mg/mL according to the PubChem database (Kim et al. 2025).

**Fig. 4:**
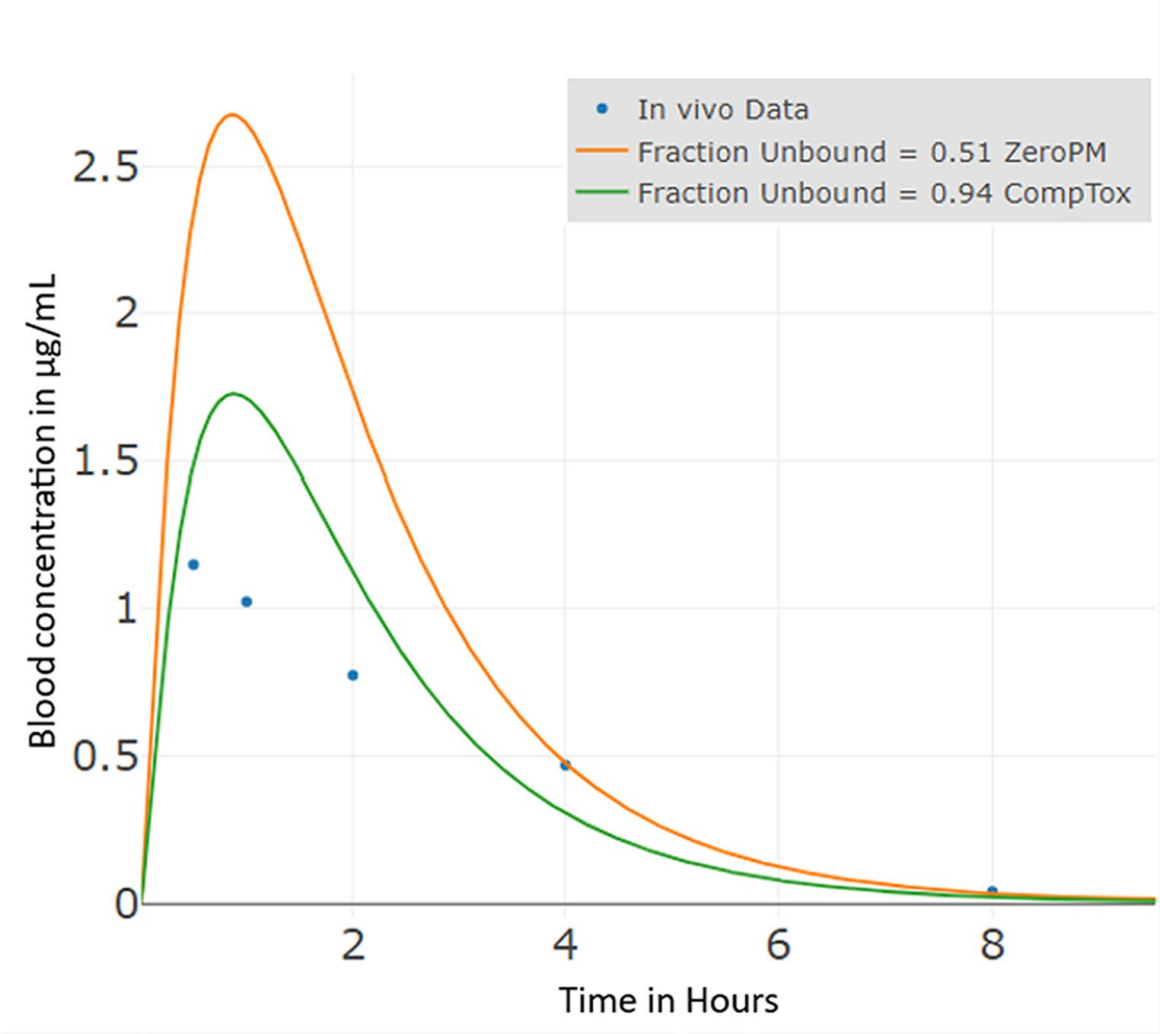
Bottom-up simulated cyromazine rat oral route model plasma concentration curve (*Clint* = 0.9 µL/min/10^6 cells) and observed in vivo plasma concentration for 3 mg/kg bw input oral dose. The observed variation in C_max_ resulted from differences in measured fub values. (Table 6)

The in vivo data accounts for the total radioactivity of the cyromazine administered in the rat study, including both the parent compound and its metabolites. The observed blood–plasma concentration–time curve (Fig. 4) provides a suitable benchmark: given the low metabolic clearance of cyromazine (Table 6), model predictions for C_max_ and T_max_ are within about two-fold of the in vivo measured kinetic values (Table 7) .

The *Clint* values, along with the *fub* of cyromazine, significantly impact the model’s output, reinforcing its classification as a low clearance compound (Esaki et al. 2019). The apparent permeability (*Papp*) values presented in ‘Table 6’ further illustrate the low to medium level of absorption for cyromazine (Fig. 4).

**Table 8:** Cyromazine PBK model results – Ratio of the PBK model prediction to in vivo measured concentration

Data type	PBK Model prediction		In Vivo Data (EFSA DAR (2007)- Cyromazine)
	fub = 0.51 (factor)	fub = 0.94 * (factor)	
C_max (µg/ml)	2.67 (2.3)	1.73 (1.5)	1.15
T_max (h)	0.86 (1.7)	0.88 (1.7)	0.50
AUC (µg/mL * h)	6.88 (1.5)	4.44 (1.0)	4.41

* CompTox dashboard (Williams et al. 2017)

A factor (indicated in brackets) is calculated as the ratio of model predictions to the measured in vivo data to assess the PBK model’s accuracy and performance through goodness of fit

The intrinsic hepatic clearance value used in modelling is 0.9 µL/min/10^6 cells. Based on the data comparison, the model with *fub* = 0.94 yields significantly better results than the model with *fub* = 0.51 when compared to in vivo data (EFSA DAR (2007)- Cyromazine). While both models use the same intrinsic clearance value, the higher fraction unbound parameter produces more accurate predictions.

The *fub* = 0.94 model shows better fit when compared with the rat measured in vivo kinetic data. C_max_ is better predicted, though both models still overpredict the maximum concentration. T_max_ shows similar overprediction in both scenarios. Most notably, AUC shows significant improvement with *fub* = 0.94, achieving nearly perfect agreement with in vivo data.

### Sensitivity analysis

**Fig. 5:**
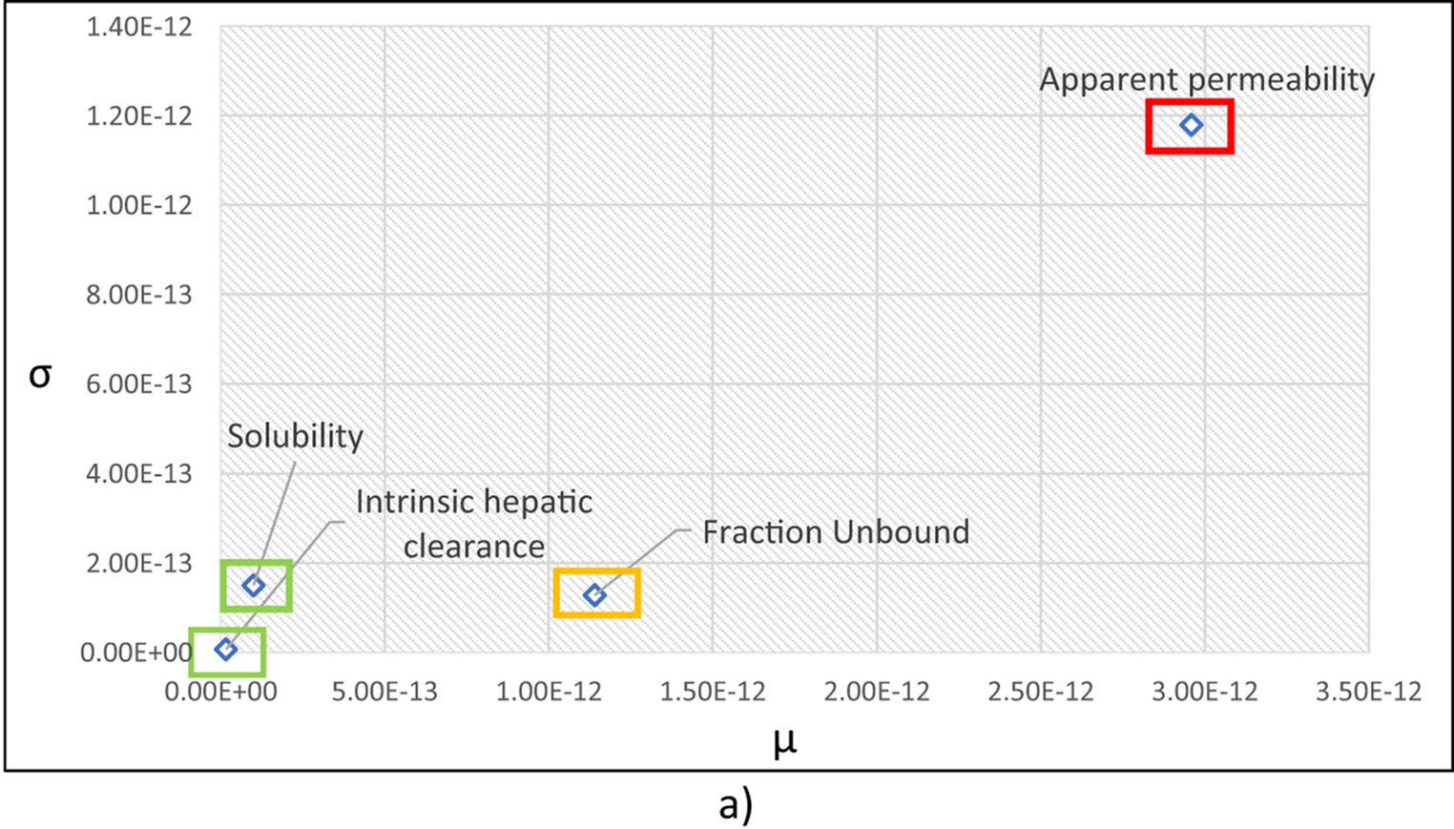

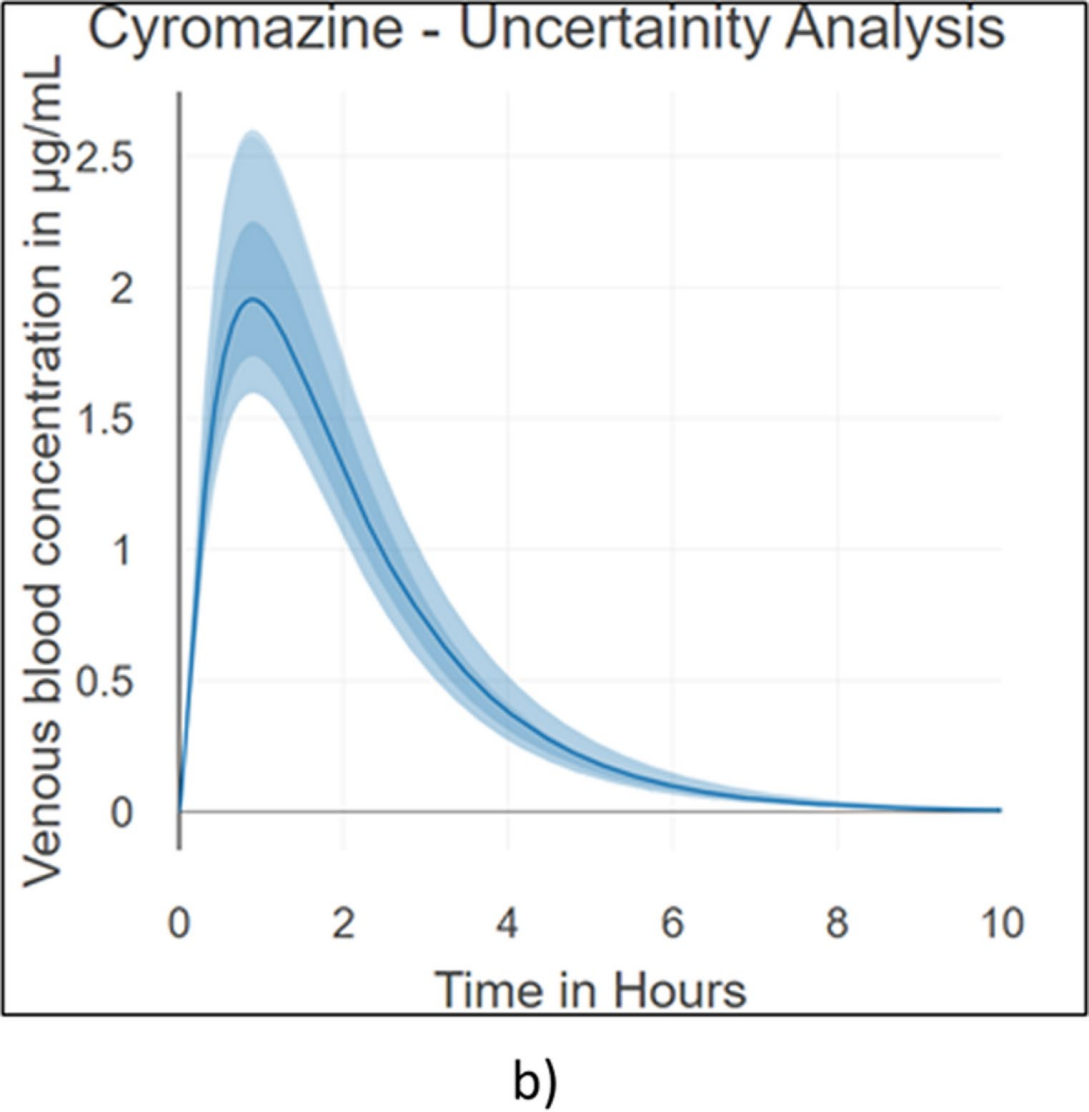
Cyromazine **a** Sensitivity analysis by Morris plot** b** Uncertainty analysis with compound specific parameters within 2-fold range

Figure 5a presents a Morris global sensitivity plot (Covington and Gearhart 2020; Paini et al. 2021; Najjar et al. 2022) for the model outputs. The x-axis (µ) is the mean of the elementary effects and reflects each ADME parameter’s overall influence on the outcome, while the y-axis (σ) is the standard deviation of the elementary effects and captures nonlinearity and interactions among all four ADME parameters.

Using Morris screening plots and MCMC analyses (Covington and Gearhart 2020; Paini et al. 2021), the sensitivity ranking in descending order is: apparent permeability > maximum solubility > fraction unbound > intrinsic hepatic clearance. Similar to triazoles, triazines also exhibit low intrinsic hepatic clearance *(Clint)*, which is reflected in the sensitivity analysis (Fig. 5a) as the ADME parameter with the smallest impact. For cyromazine, although the fraction unbound contributes to sensitivity, it is not the most sensitive parameter, since its value is very high (*fub* = 0.94; Table 3). This makes the apparent permeability *(Papp)* the most sensitive parameter.

Figure 5b shows the MCMC-based uncertainty for venous blood concentration over time, where the solid line represents the posterior mean, the darker band is the central credible interval, and the lighter band is a wider credible interval. Uncertainty is highest near the absorption peak (around C_max_) and steadily decreases as elimination proceeds.

Using simplified assumptions, the bottom-up PBK model can estimate the bioavailable plasma concentrations (Table 8). Additionally, the read-across method is employed to parameterize various data-poor compounds within the triazine group (Supplementary Sect. 1).

### Triazole / Triazine human extrapolation

The human PBK model uses the same assumptions as the validated rat model. It predicts the bioavailable concentration of triazoles or triazines in plasma for an average human person (70 kg male) being continuously exposed to different external concentrations over 5 days (Table 3): (i) maximum water solubility (Supplementary Sect. 1), taken as the highest plausible concentration in drinking water under worst-case exposure conditions. (ii) surface and groundwater concentrations (Supplementary Sect. 1) from EMPODAT Norman database (2020) or (iii) LOAELs derived from the in vivo studies (Supplementary Sect. 4) allometrically scaled to humans (Crowell et al. 2011).

**Fig. 6:**
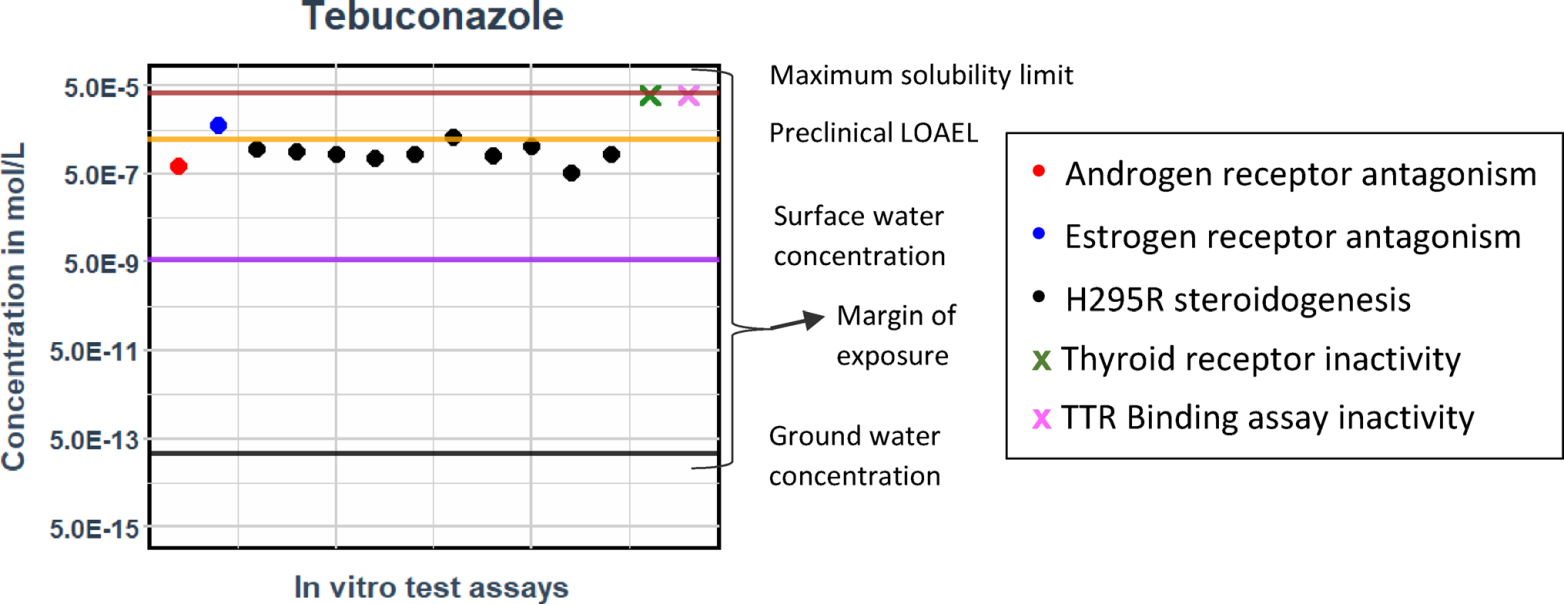
Free unbound steady state plasma concentrations (C_max_) for tebuconazole vs. free medium in vitro benchmark concentration concentrations

The estimated unbound steady state C_max_ plasma concentrations from 5 days continuous exposure are compared to the free medium in vitro benchmark concentrations from different in vitro assays described by Carlier et al. (2024) from Table 4 (also Supplementary Sects. 3 and 4). The comparison indicates that the blood - plasma concentration of tebuconazole, resulting from prolonged exposure to the LOAEL concentration, falls within the same range, approximately 1E-6 of the free medium concentrations observed in the in vitro test assays (Fig. 6). The complete set of comparisons is provided in ‘Supplementary Sect. 4’.

### Tebuconazole (Triazole) – Quantitative in vitro to in vivo extrapolation (QIVIVE)

Figure 6 represents the free medium in vitro benchmark concentration values of all in vitro assays (as dots or crosses), while the estimated blood - plasma concentrations (C_max_) from external doses are shown as horizontal lines. For tebuconazole, the AR, ER, and H295R steroidogenesis showed an activity, while the TR and TTR binding assays did not show response at the maximum tested non-cytotoxic concentration of 30 µM (Carlier et al. 2024).

Tebuconazole, a data-rich triazole, is modelled based on continuous exposure over 5 days (Table 5), using ‘Eq. 11’. The horizontal lines represent the free unbound maximum plasma concentration (C_max_) resulting from different oral input doses in the bottom-up PBK model. The black, purple, and brown lines correspond to continuous exposures at groundwater concentration, surface water concentration, and the maximum water solubility limit of tebuconazole, respectively. The orange line indicates the free unbound maximum plasma concentration derived from continuous exposure at the allometrically scaled preclinical LOAEL value.

For Tebuconazole, the in vitro assays yielded BMC values within the same range as the maximum free plasma concentration determined from the allometrically corrected LOAEL of the rodent study. The limited in vitro test battery thus provided a protective BMCs, which can be used to estimate external human doses by reverse dosimetry.

Furthermore, the comparison of blood - plasma concentrations from surface and groundwater with the in vitro BMC values/or LOAELs revealed a margin of exposure (MoE) of 10^2 times and 10^6 times respectively. This finding indicates that the Tebuconazole concentrations found in surface and ground water are safe and protective based on both the in vitro and in vivo data. (Fig. 6).

### QIVIVE of triazoles

**Fig. 7:**
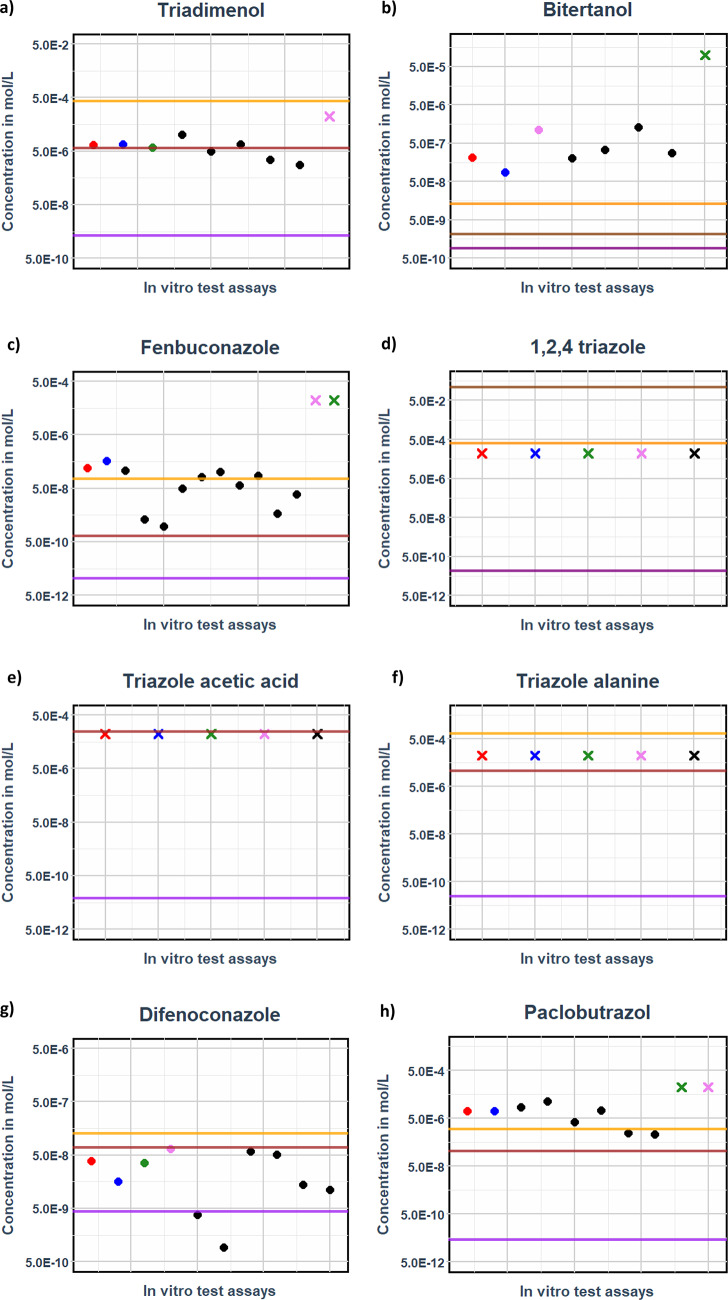

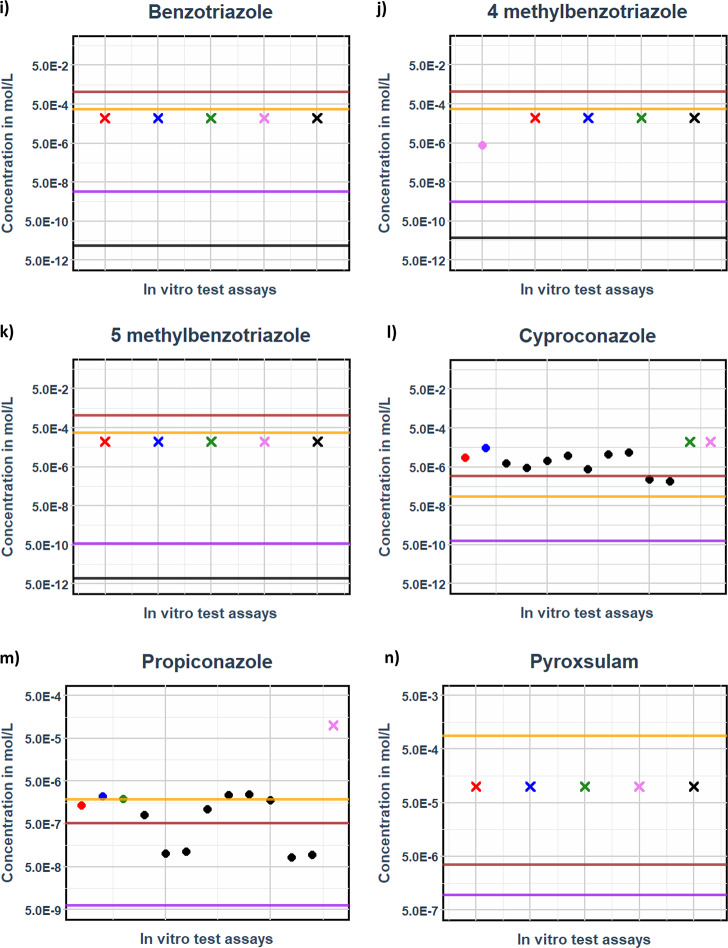
This figure compares free unbound steady-state plasma concentrations of triazole analogues from drinking water exposure. Horizontal lines (**--**) represent LOAEL-derived concentration (orange), maximum water solubility (brown), surface water (purple), and groundwater (black), while dots (•) and crosses (**X**) denote in vitro benchmark concentrations for activity (EC20) and inactivity (IR1.5), respectively. This analysis is conducted through read-across ADME parameterization, illustrating the relationship between exposure levels and their corresponding biological effects

Fig. 6 and Fig. 7 demonstrate that among the 16 selected triazoles, 8 compounds exhibited activity in one or more assays, while the remaining 8 were inactive. For the active triazoles, the free benchmark concentrations are protective, as they fall within the same range as the PBK model-derived unbound LOAEL Cmax values. This finding holds true for all compounds except bitertanol. In contrast, the 8 inactive compounds showed unbound LOAEL Cmax values at substantially higher concentrations than the maximum tested in vitro benchmark concentration.

From’ Fig. 6’ and ‘Fig. 7’ all the compounds indicate the ‘margins of exposure’ between the ground and surface water unbound C_max_ values to the LOAEL- derived unbound C_max_. The margin of exposure between the unbound surface water C_max_ and the unbound LOAEL C_max_ consistently exceeded 10^2 mol/L for all compounds, except for bitertanol and difenoconazole.

### **Cyromazine (triazine) – Quantitative in vitro to in vivo extrapolation (QIVIVE)**

**Fig. 8:**
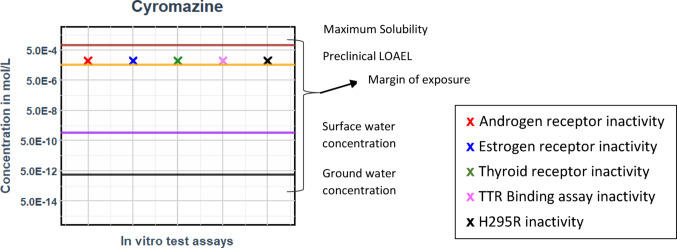
Free unbound steady state plasma concentrations of the drinking water exposure for cyromazine vs. free medium in vitro benchmark concentration comparison (No in vitro response at the maximum measured non-cytotoxic concentration − 100 µM)

Cyromazine, a data-rich triazine, is modelled based on continuous exposure over 5 days using ‘Eq. 11’, similar to tebuconazole (Table 5). At the highest measured non-cytotoxic concentration of 100 µM, cyromazine exhibits no response in any of the in vitro test assays (Fig. 8) (Carlier et al. 2024). The in vivo preclinical LOAEL values (EFSA DAR (2007)- Cyromazine) are around 10-fold higher than the highest measured non-cytotoxic concentration that exhibited no specific response.

**Fig. 9:**
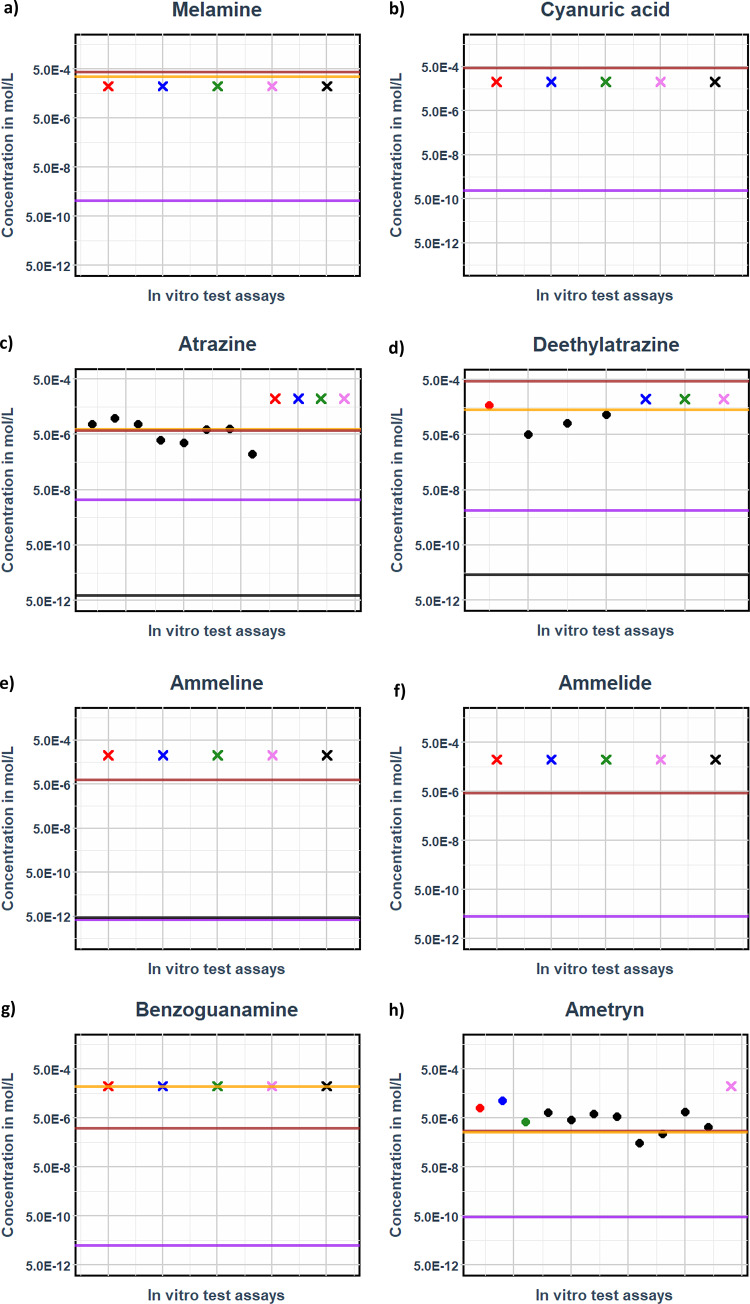
The figure compares free unbound steady-state plasma concentrations of triazine analogues from drinking water exposure. Horizontal lines (**--**) represent LOAEL-derived concentration (orange), maximum water solubility (brown), surface water (purple), and groundwater (black), while dots (•) and crosses (**X**) denote in vitro benchmark concentrations for activity (EC20) and inactivity (IR1.5), respectively. This analysis is conducted through read-across ADME parameterization, illustrating the relationship between exposure levels and their corresponding biological effects

Of the 9 triazines tested, only 3 namely atrazine (d), deethylatrazine (e), and ametryn (h)—showed activity at the highest tested concentrations (Figs. 8 and 9). These in vitro-derived benchmark concentrations are considered protective, as they align closely with the unbound LOAEL C_max_ values predicted by the PBK model.

From’ Fig. 8’ and ‘Fig. 9’ all the compounds indicate the ‘margins of exposure’ between the ground and surface water unbound C_max_ values to the preclinical LOAEL- derived unbound C_max_. The LOAEL-derived unbound C_max_ (brown horizontal line) values consistently exceeded surface water-derived unbound C_max_ values (purple horizontal line) by more than two orders of magnitude (> 10^2), resulting in margins of exposure that uniformly surpassed by a factor of 100.

## Discussion

This study successfully developed and validated a QIVIVE framework for two classes of PM compounds: triazoles and triazines. The case studies demonstrate that simple bottom-up PBK models, parametrized using few compounds specific in vitro ADME parameters are effective for modelling the bioavailability of these substance classes.

This is in line with earlier findings from Fragki et al. (2017), who demonstrated the application of a generic PBK modelling approach for quantitative in vitro-to-in vivo extrapolation across diverse classes of developmental toxicants. By comparing PBK-simulated plasma concentrations at toxic in vivo doses with in vitro effective concentrations, they showed that incorporating kinetics into QIVIVE provides a more biologically plausible approach. Similarly, Chan et al. (2019) reported that bottom-up PBK models built solely from in vitro transporter and metabolism data predicted human plasma exposure metrics (AUC, C_max_, clearance) within two-fold of observed clinical value, while Strikwold et al. (2017) demonstrated that IVIVE-based PBK models accurately estimated in vivo points of departure for phenols, differing by less than 3.6-fold from experimental animal data.

The article from Punt et al. (2022) evaluates the predictive performance of generic human physiologically based kinetic (PBK) models using only in vitro and in silico input data to predict plasma C_max_ concentrations, aiming to enable quantitative in vitro-to-in vivo extrapolation (QIVIVE) without relying on animal in vivo data for next-generation risk assessment. The study found that 77% of compounds (34 out of 44) could be predicted within 5-fold accuracy when using optimal input approaches, specifically in vitro hepatic clearance measurements, the Rodgers and Rowland method (Rodgers et al. 2005; Rodgers and Rowland (2006) for tissue to plasma partition coefficients, and the Lobell and Sivarajah method for fraction unbound in plasma. This is in accordance with our case, where the bottom-up PBK model predicted C_max_ and T_max_ to be within 2-fold to the rat in vivo situation (Tables 7 and 8).

Our approach resulted in a proof of concept with one data rich compound with the extrapolation from rat to human for each class and further extrapolated the modelling across the group using a read-across approach. This is in accordance with Hines et at. (2021) where nicotine is used as model compound for rat to human extrapolation. Similarly, the uncertainty of the PBK model is quantifiable in comparison to one in vivo ADME dataset, and major differences within the grouped compounds could be detected from the measured in vitro ADME properties. This is in accordance with Sundqvist et al. (2015) in which one ‘data rich’ compound is modelled in a PK/PD framework to estimate human dose and its associated uncertainty using in vitro (hepatocyte) data, then later compared with additional similar compounds to evaluate the consistency and applicability of the dose estimation approach.

Our comparison revealed no major differences between experimentally measured in vitro ADME parameters (*Papp*,* Clint*,* fub*,* b/p* ratio) and values retrieved from the CompTox dashboard (Williams et al. 2017), indicating low parameter uncertainty for triazoles and triazines. This concordance supports the conclusion that a simplified bottom-up PBK model structure is adequate for QIVIVE purposes for these compound classes. However, compound-specific variability in ADME parameter accuracy should not be overlooked. Punt et al. (2023) reported substantial discrepancies between experimentally determined and literature-based values for other compounds, with differences in *Clint* and *Papp* reaching approximately 20-fold and 5-fold, respectively.

From the sensitivity analysis, apparent permeability (*Papp*) is the most sensitive parameter for cyromazine. *Papp* values vary across the measured compounds: cyromazine and atrazine are on the order of *Papp* * 1E-6 cm/s in the range of moderate to low absorption, while melamine is around *Papp* * 1E-7 cm/s contributing to the low absorption range. This relative low absorption in triazines is attributed to their low logPow values and low molecular weights in triazines. This is in accordance with the ranges of apparent permeability *(Papp)* in Kus et al. 2023 and Pham-The et al. 2013.

However, several compounds, including triazole acetic acid and ammelide, showed *Papp* below the limit of detection (Supplementary Sect. 1). This introduced uncertainty in parameter estimation. To address this, we employed a worst-case read-across approach, assigning conservative *Papp* values for data-poor compounds (Supplementary Sect. 1). This strategy was also used by Fowler et al. (2022) when facing similar detection challenges. It ensures protective risk estimates despite data gaps. However, it may increase uncertainty ranges and produce more conservative benchmark dose predictions for compounds with limited permeability data.

In contrast to cyromazine, sensitivity in tebuconazole is driven by the fraction unbound *(fub)* due to its lower value. However, no consistent trend in fraction unbound was observed across triazoles. In general, compounds with higher logPow and higher molecular weight tend to exhibit *Papp* (Supplementary Sect. 1). The common observation is that for triazoles and triazines the *Clint* value is low to moderate. This is in accordance with Aggarwal and Sumran (2020) and Lu et al. (2023) which triazoles and triazines are the more stable compounds and are difficult to cleave.

In the ZeroPM project, we propose a risk-based prioritization matrix specifically designed for persistent (P) and mobile (M) compounds (Gouin et al. 2024), tailored to address the persistent and mobile compounds. This prioritization framework is particularly beneficial for our target classes of triazoles and triazines, which are identified as representative classes of persistent and mobile compounds. However, it is important to note that this approach is currently limited to these classes of compounds (triazoles and triazines), and further studies are necessary to extend the framework to the compounds with similar properties.

The QIVIVE approach is essential to derive meaningful comparisons of hazardous concentrations found in in vitro tests with human exposure concentrations in drinking water. The in vitro test assessment conducted by Carlier et al. (2024) applied a set of assays tailored to mainly endocrine endpoints. 6 triazines and 7 triazoles were inactive in all tested assays, indicating that the biological coverage of the applied testing battery was limited.

Triazoles are known to activate nuclear receptors FXR, CAR and PXR leading to liver toxicity which progresses to cancer formation after chronic life-time exposure (Goetz and Dix 2009; Heise et al. 2018; Hester et al. 2012). Receptor activation leads to CYP enzyme induction, impairment of lipid and steroid homeostasis and an increase in cell proliferation, with prolonged chronic cell proliferation being discussed as one reason for triazoles to cause liver cancer (Goetz and Dix 2009; Heise et al. 2018). Chronic effects are a challenge for in vitro assays, as typical in vitro exposure scenarios are limited to a short-term exposure of one to a few days.

The in vitro assay battery could be extended by e.g. transcriptomics or reporter assays in relevant in vitro systems like (primary) human hepatocytes to capture a broader spectrum of biological processes including e.g. key events related to liver toxicity such as hypertrophy, fatty degeneration and effects on lipid and steroid homeostasis. Other projects like RISK-HUNT3R and APCRA are currently working on the development of tiered testing and assessment strategies for the endpoint systemic toxicity (Leist et al. 2025).

Tools like bottom-up PBK models are essential for reducing animal testing, addressing the ethical concerns associated with sacrificing large numbers of animals in in vivo studies. Validation of our approach using in vivo data from data-rich compounds provides confidence for applying it to data-poor compounds through read-across methods. However, uncertainties remain since direct in vivo comparisons are not feasible for data-poor compounds. Similar uncertainties in bottom-up PBK approaches were also reported by Punt et al. (2022). This must be solved with further extensive in vitro based ADME measurements and testing which has be addressed by the scientific community.

## Conclusion

In conclusion, this study successfully establishes a quantitative in vitro to in vivo extrapolation (QIVIVE) framework for assessing the bioavailability of triazoles and triazines, highlighting the effectiveness of integrating physiologically based kinetic (PBK) modelling and read-across methodologies for chemical prioritization purposes in risk assessment workflows. The findings underscore the critical role of the bottom-up PBK model with in vitro derived ADME parameters such as the fraction unbound *(fub)*, apparent permeability *(Papp)* and intrinsic hepatic clearance *(Clint)* in predicting human internal concentrations from environmental doses, emphasizing the need for accurate measurements in future research. This work not only addresses the growing concerns regarding the environmental impact of these persistent and mobile compounds but also creates a framework for prioritizing other similar compounds of PM class.

The QIVIVE approach using PBK modelling bridges the gap between in vitro test assays with specific endpoints and human internal concentrations resulting from exposure to drinking water contaminants. The integration of new approach methodologies (NAMs) into this framework represents a significant advancement in toxicology, reducing reliance on animal testing while still providing essential data for risk-based prioritization for regulatory purposes. By employing a combined approach that integrates in silico and in vitro models with read-across methodology, this study demonstrates how comprehensive risk-based prioritization can be achieved.

A simple bottom-up ADME-parameterized PBK model was used to predict human internal bioavailable concentrations. For data-poor compounds, a read-across approach was employed, guided by sensitivity analysis. Where data for sensitive parameters were lacking, we applied a worst-case approach to derive conservative estimates of the bioavailable fraction. This intentional overestimation ensures more protective chemical prioritization and risk assessment.

The QIVIVE comparisons yielded, that the in vitro free benchmark concentrations (EC20 and IR1.5) are protective in nature when compared with the LOAEL derived unbound C_max_ as they align closely with the unbound LOAEL C_max_ values. The unbound C_max_ plasma concentrations derived from surface and ground water has the margins of exposure atleast 100 times lower than the in vitro benchmark concentrations and the LOAEL derived unbound C_max_.

Future research should focus on refining input parameters and expanding the database of in vitro absorption, distribution, metabolism, and excretion (ADME) data to enhance the accuracy and reliability of predictions for a broader range of persistent and mobile compounds. This research not only advances our understanding the toxicokinetics and toxicodynamics of triazoles and triazines but also paves the way for more effective regulatory frameworks aimed at mitigating the risks associated with persistent and mobile chemicals in the environment, thereby ensuring better protection with respect to environment and human health. We need more such case studies to build and increase confidence in this NAMs based approach and to learn and address its limitations.

## Supplementary Information

Below is the link to the electronic supplementary material.

Supplementary Material 1

Supplementary Material 2

Supplementary Material 3

Supplementary Material 4

Supplementary Material 5

## Data Availability

All data supporting the findings of this study are available in the Supplementary Material, provided as Excel files (.xlsx) in accordance with FAIR data principles. The supplementary sections include: compound selection and ADME parameters (Supplementary Sect 1); VIVD model physicochemical parameters and culture conditions (Supplementary Sect 2); model corrections for various assays including AR, ER, TR, TTR, H295R, and IR1.5 (Supplementary Sect 3); environmental exposure data via drinking water and LOAEL values (Supplementary Sect 4); and corrected concentrations with cellular partitioning data for all tested assays (Supplementary Sect 5). These data have been made accessible in accordance with FAIR (Findable, Accessible, Interoperable, and Reusable) data principles to ensure transparency and reproducibility of the research findings.
